# FASTINA – protocol for a randomised feasibility trial of time-restricted eating to improve response to multiple myeloma therapy

**DOI:** 10.1186/s40814-026-01914-7

**Published:** 2026-09-03

**Authors:** Anna Franziska Hamm, Jan Vorwerk, Mona Maria Merz, Martin Smollich, Christina Schwitlick, Kay Horn, Inke Regina König, Theo Leitner, Lorenz Oelschläger, Helal Ahmed, Felix Sommer, Nikolas von Bubnoff, Cyrus Khandanpour

**Affiliations:** 1 Department of Haematology and Oncology, University Cancer Centre Schleswig-Holstein (UCCSH), University Hospital Schleswig-Holstein (UKSH) – Campus Lübeck, Ratzeburger Allee 160, Lübeck, 23538 Germany; 2 Detlef Zillikens Clinician Scientist Academy, University of Lübeck, Ratzeburger Allee 160, Lübeck, 23538 Germany; 3 Institute of Nutritional Medicine, University Hospital Schleswig-Holstein (UKSH) – Campus Lübeck, Ratzeburger Allee 160, Lübeck, 23538 Germany; 4 Zentrum Für Klinische Studien (ZKS), University of Lübeck, Marie-Curie-Straße 1, Lübeck, 23562 Germany; 5 Institute of Medical Biometry and Statistics, University of Lübeck, Ratzeburger Allee 160, Lübeck, 23538 Germany; 6 Department of Haematology and Oncology, Klinikum Oldenburg, Rahel-Straus-Straße 10, Oldenburg, 26133 Germany; 7 University Medicine Oldenburg (UMO), Carl Von Ossietzky University of Oldenburg, Ammerländer Heerstraße 114–118, Oldenburg, 26129 Germany; 8 Institute of Clinical Molecular Biology, University of Kiel and University Hospital Schleswig-Holstein (UKSH) – Campus Kiel, Rosalind-Franklin-Straße 12, Kiel, 24105 Germany

**Keywords:** Multiple myeloma, Time-restricted eating, Fasting, Minimal residual disease

## Abstract

Multiple myeloma (MM) is an incurable plasma cell malignancy with relapses and diminishing response durations. Preclinical studies suggest that time-restricted eating (TRE)-related metabolic changes may enhance chemosensitivity by exploiting cancer cell vulnerabilities to nutrient deprivation. Clinical data in solid tumours remain limited and heterogeneous, mainly supporting feasibility and possible toxicity reduction rather than definitive efficacy. While efficacy has been explored in breast cancer patients, there is a lack of data for haematological patients, including MM. FASTINA (Fasting in Staging of MM – Intervention for Nutritional Adherence) will therefore primarily assess feasibility and safety and will explore preliminary efficacy of incorporating TRE into standard MM therapy. FASTINA is a single-centre, open-label, parallel-group randomised controlled feasibility trial (DRKS00034370). 54 adults with MM will be randomised 1:1 to either 16:8 TRE + nutritional counselling or nutritional counselling alone for 12 weeks during systemic therapy. Primary endpoints are adherence to the diet (adherence ≥ 80%) and complete response with measurable residual disease (MRD) negativity at treatment end. Secondary endpoints include response rates, nutritional status, body composition changes, frequency of ≥ grade 3 adverse events (AEs), progression-free (PFS) and exploratory overall survival (OS), quality of life (QoL), and fatigue. FASTINA will evaluate whether TRE is feasible and safe in selected MM patients and whether there is a hypothesis-generating signal for improved treatment response, informing the design of a subsequent phase II trial.

**Trial registration** Deutsches Register Klinischer Studien (DRKS), DRKS00034370. Registered 21 June 2024, https://drks.de/search/de/trial/DRKS00034370.

**Supplementary Information:**

The online version contains supplementary material available at https://doi.org/10.1186/s40814-026-01914-7.

## Introduction

Multiple Myeloma (MM) is a clonal plasma cell malignancy with a relapsing–remitting course and therapy resistance. Despite advances, MM remains incurable [23]. Standard treatments include corticosteroids, immunomodulatory drugs (IMiDs), proteasome inhibitors (PIs), monoclonal antibodies (mABs), autologous stem cell transplants (ASCT), and CAR T-cell therapies. Poor long-term prognosis, especially after the first relapse, necessitates supportive strategies to improve outcomes.

**Fig. 1:**
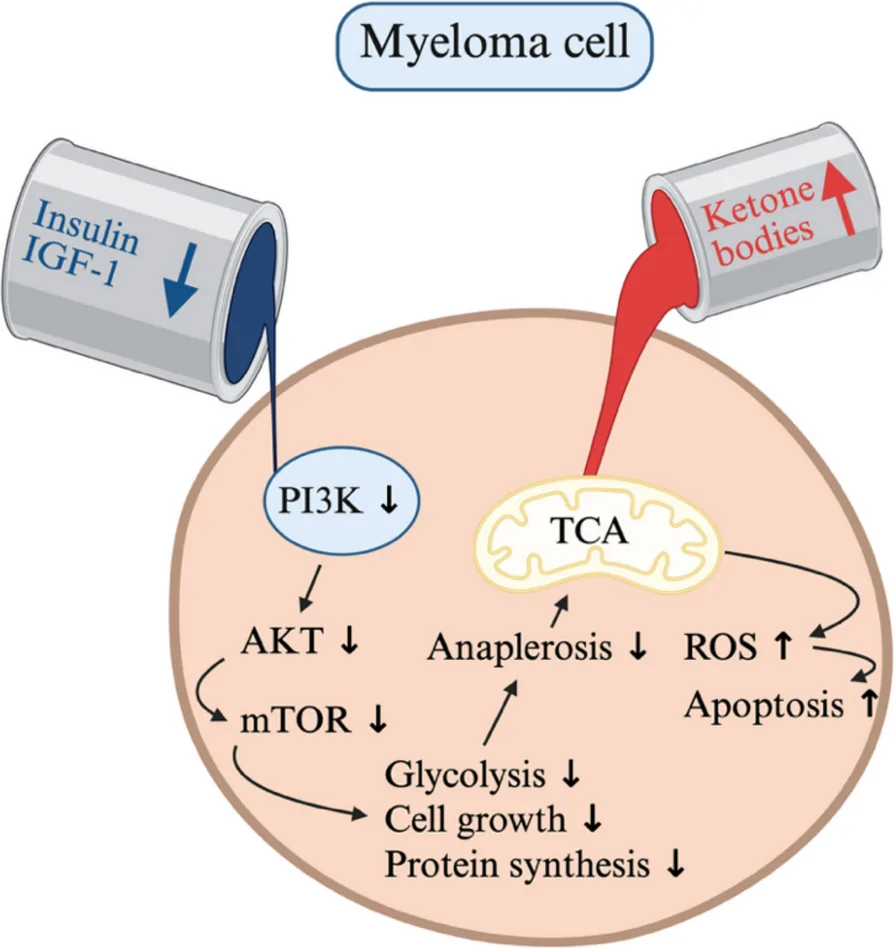
Hypothesised fasting-induced metabolic switch in Multiple Myeloma (MM) cells. Time-restricted eating (TRE) reduces systemic insulin and IGF-1 signalling, potentially decreasing PI3K/AKT activity and mTOR pathway signalling. In parallel, TRE may increase circulating ketone bodies, which can fuel the tricarboxylic acid cycle (TCA) and influence reactive oxygen species (ROS) production. These proposed metabolic changes hold the potential to sensitise MM cells to therapy or impair growth and survival. Adapted from Dal Bello [7] and Luda [19]. Designed with BioRender (Toronto, ON, Canada)

Nutritional and metabolic states influence cancer therapy and tumour biology. Dietary components like vitamin C or green tea polyphenols can interfere with anti-MM drugs by reducing PI activity [13, 36]. Beyond nutrients, the metabolic state from caloric restriction may modulate drug absorption and treatment response [25, 33]. FASTINA (Fasting in Staging of MM – Intervention for Nutritional Adherence) specifically evaluates the feasibility of TRE, with nutritional counselling intended to maintain caloric and protein intake during therapy. Fasting and TRE are potential adjuncts due to their metabolic and cellular stress effects [9, 18]. Preclinical studies suggest that nutrient deprivation can decrease insulin and IGF-1 signalling, lower glucose and growth factor levels, increase ketone bodies, and activate autophagy [1, 2] (Fig. 1). This concept of differential stress resistance suggests fasting may protect normal tissues from chemotherapy toxicity while sensitising tumour cells to treatment [15, 26]. Short-term fasting may inhibit pro-growth pathways via ketone-mediated mechanisms, thereby promoting autophagic clearance of damaged cells and impairing tumour cell survival [12]. Fasting may also enhance cytotoxic T-cell activity and reduce immunosuppressive regulatory T-cells [12]. These insights provide a rationale to investigate whether fasting-related interventions can augment tumour kill or reduce toxicity, while recognising that the clinical magnitude and relevance remain uncertain [5, 21]. Pilot studies in solid tumours support the feasibility and safety of short-term fasting during chemotherapy and suggest a potential to reduce toxicity [3, 8, 10, 30], but randomised data remain limited, adherence can decline during repeated cycles, and effects on pathological complete response or severe toxicity have been inconsistent [11, 32].

However, TRE is understudied in patients with haematological malignancies such as MM, and the available clinical evidence largely derives from short-term fasting or FMD approaches in solid tumours rather than prolonged daily TRE during intensive MM treatment. Many MM patients receive prolonged therapy and high-dose steroids affecting glucose and IGF-1 levels. Dexamethasone may attenuate fasting-induced reductions in insulin/IGF-1, stimulate appetite, induce hyperglycaemia, and disturb circadian metabolic rhythms. Therefore, steroid timing and dose may confound biological effects attributed to TRE [10, 14, 20]. Nonetheless, as MM remains incurable and deeper responses including MRD negativity are clinically relevant, carefully monitored metabolic-supportive strategies merit prospective evaluation, provided that safety and nutritional adequacy are prioritised. FASTINA addresses this gap by examining if TRE during MM therapy is feasible. We hypothesise that TRE is well-tolerated in MM patients and could deepen the haematologic response.

## Methods

### Trial design

FASTINA is an open-label, parallel-group, single-centre randomised feasibility trial evaluating TRE during standard MM therapy. 54 patients at the University Hospital Schleswig–Holstein (UKSH) – Campus Lübeck, will be randomised 1:1 to two arms: Arm A (experimental): 16:8 TRE (daily 16 h fasting/8 h eating window) for 12 weeks in addition to standard nutritional counselling. Arm B (control): Standard nutritional counselling alone for 12 weeks (no fasting intervention). The 16:8 schedule was selected as a pragmatic compromise between a fasting interval long enough to induce daily metabolic switching and an 8 h eating window that still permits patients to meet energy and protein goals during therapy. More restrictive prolonged fasting was avoided for safety reasons.

**Fig. 2:**
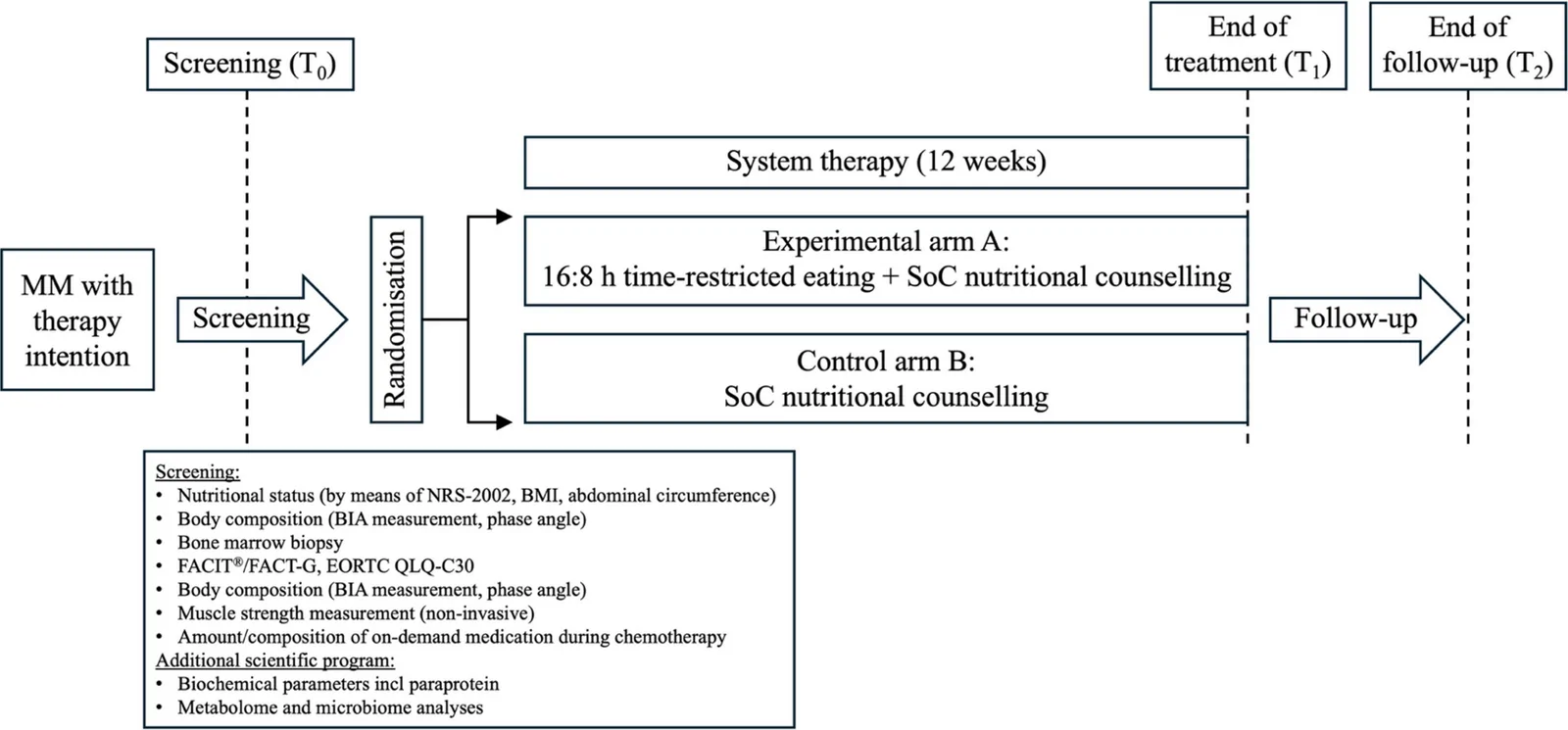
FASTINA trial schema. Adults with multiple myeloma (MM) scheduled for therapy undergo screening (T_0_; ≤ 28 d) and are randomised 1:1 to experimental arm A (16:8 h time-restricted eating (TRE) + standard-of-care (SoC) nutritional counselling) or control arm B (SoC nutritional counselling alone) during 12 weeks of therapy. Assessments occur at the end of treatment (T_1_) and after a 12-week follow-up (T_2_). Abbreviations: BIA: bioelectrical impedance analysis; BMI: body mass index; EORTC QLQ-C30: European Organisation for Research and Treatment of Cancer Quality of Life Questionnaire-Core 30; FACIT®: Functional Assessment of Chronic Illness Therapy; FACT-G: Functional Assessment of Cancer Therapy-General; MM: multiple myeloma; NRS-2002: Nutritional Risk Screening 2002; SoC: standard of care; T_0_: screening; T_1_: end of treatment; T_2_: end of follow-up

All participants continue their standard-of-care anti-MM therapy per guidelines. Trial duration is ≈ 7 months, including 28 d screening, a 12-week intervention during active MM therapy, and 12-week follow-up. Key time points are baseline (T_0_), end of the 12-week intervention (T_1_), and end of the 12-week follow-up (T_2_) (Fig. 2).

The trial is open-label as blinding of TRE intervention is not possible. Objective endpoints such as minimal residual disease (MRD) assessments will be evaluated blinded to minimise bias. Randomisation is performed to ensure allocation concealment. Stratified randomisation will balance groups for key factors (line of therapy, baseline BMI < 25/≥ 25 kg/m^2^, and gender).

### Participants

The trial includes adults (≥ 18 years) with confirmed MM starting a new line of systemic therapy (first-line or relapsed) per International Myeloma Working Group (IMWG) criteria. Patients must receive standard anti-MM therapy and be medically fit (Eastern Cooperative Oncology Group status 0–2, or 3 if solely due to MM symptoms).

Major exclusion criteria include conditions incompatible with caloric restriction, unintended weight loss, or malnutrition (BMI < 20 kg/m^2^, or cachexia defined as > 5% weight loss in 6 months or > 10% in 12 months) [28], recent major treatments (ASCT within 100 d), history of eating disorders or severe psychiatric illness, and requirement for nutritional support. Active, uncontrolled medical conditions (e.g., insulin-dependent diabetes, severe infections, significant organ dysfunction), known HIV or active hepatitis B/C infection, and pregnancy or lactation are also exclusions. These criteria deliberately select patients without clinically relevant malnutrition or uncontrolled metabolic comorbidity. This is a safety measure for this pilot trial, which limits generalisability to frailer or cachectic MM populations.

All participants provide written informed consent approved by the central ethics committee of the University of Lübeck, compliant with GCP guidelines (2024-182_1). Participants may withdraw at any time and without giving reasons.

### Interventions

Patients randomised to arm A will undergo a 16:8 TRE schedule daily for the 12-week intervention period. The timing of the eating window is flexible but should remain consistent wherever feasible. Outside the designated 8 h window, patients abstain from foods or caloric beverages. Non-caloric fluids are allowed during fasting hours. Adherence is defined as maintaining the 16 h fasting goal on at least 80% of days, with minor deviations permitted. TRE is not intended to induce caloric restriction or weight loss. During the 8 h eating period, patients are counselled to maintain usual energy intake and adequate protein intake, with emphasis on nutrient-dense meals, symptom-adapted meal planning, and widening or pausing the fasting window if intake becomes insufficient. Patients randomised to arm B continue their normal eating pattern.

All patients receive standard nutritional counselling from a nutritionist, including individualised guidance on energy and protein intake during therapy, management of nausea, mucositis, diarrhoea, constipation, dysgeusia or reduced appetite, and healthy eating recommendations. Counselling is identical in both arms, delivered at baseline and reinforced during clinic visits. Additional nutritionist contact is triggered by weight loss, reduced intake, or symptoms compromising dietary adequacy.

All participants maintain a food diary to record eating times and protocol deviations. Arm A patients record the start and end times of their eating window. Arm B patients record their typical mealtimes. The diary also documents symptoms or adverse events (AEs). These logs are reviewed at each visit to assess adherence. To reduce reliance on self-report, adherence and nutritional adequacy will be triangulated with structured interview, body weight, BMI, NRS-2002, BIA-derived body composition angle, and metabolic biomarkers (glucose, ketones, insulin, and IGF-1) measured according to the study schedule. We recognise that biomarkers are supportive rather than definitive adherence measures as they can be influenced by treatment, steroids, and intercurrent illness.

All participants receive standard anti-MM therapy. Various standard treatments are allowed, reflecting real-world practice. Dexamethasone is given per standard dosing in both arms. Steroid dose, schedule, antidiabetic medication, and metabolic symptoms will be documented, and exploratory analyses will consider steroid-containing regimens and timing of biomarker sampling relative to dexamethasone exposure. Supportive treatments (antivirals, bisphosphonates, or transfusions) are permitted as needed. Patients who undergo stem cell harvest or transplant during the study will be handled per protocol. Use of additional dietary supplements is discouraged and must be documented. There are no prohibited medications aside from experimental metabolic therapies.

To ensure patient safety, predefined criteria trigger temporary pause, adaptation, or discontinuation of TRE. Weight loss > 5% from baseline, worsening NRS-2002 score, symptomatic hypoglycaemia, severe dehydration, or clinically relevant electrolyte/metabolic disturbance will prompt investigator and nutritionist review and may lead to temporary widening of the eating window or pausing of TRE. The intervention will be discontinued if arm A patients lose > 10% of baseline body weight or if clinically significant AEs are judged to be related to TRE. Significant AEs will prompt evaluation by the DMC and could lead to modification or termination of the intervention. Patients in arm A who discontinue the fasting protocol continue in the trial for outcome assessment, and their data will inform feasibility results (intention-to-treat (ITT) analysis).

### Trial status

Recruitment began in July 2024 and is ongoing. At the time of submission to *Pilot and Feasibility Studies*, 8 patients have been randomised. Based on an expected recruitment rate of approximately 10 participants per year, recruitment is anticipated to be completed by December 2029. Recruitment rate, screen-failure reasons, refusal, dropout, and non-adherence will be reported as feasibility outcomes. If the predefined safety and adherence criteria are met, a multicentre phase II trial will be pursued to improve recruitment, external validity and statistical power.

### Progression criteria

Progression to a definitive randomised efficacy trial will be considered if the pilot study demonstrates: (I) recruitment of at least 80% of the planned sample within the recruitment period, (II) retention of ≥ 85% of participants until the primary endpoint, (III) adherence to the time-restricted eating intervention in ≥ 75% of intervention participants, (IV) ≥ 90% completeness of key clinical and patient-reported outcome data, (V) no unexpected intervention-related safety concerns, and (VI) overall acceptability of study procedures for participants and investigators. These criteria will be interpreted collectively to determine whether modifications are required before initiating a full-scale efficacy trial.

## Outcomes

### Primary outcomes

Adherence rate: Proportion of patients in the TRE arm adhering to the 16:8 schedule on ≥ 80% of days over the 12-week intervention, reported with a 95% confidence interval (CI).

Therapy response (CR/MRD rate at end of treatment): The combined rate of CR and/or MRD negativity in bone marrow (BM) at end of therapy. CR is defined by IMWG criteria, and MRD negativity at a sensitivity of 10^–5^ by fluorescence-activated cell sorting (FACS) or next-generation sequencing (NGS) on BM aspirate. The fraction will be compared between arms as an exploratory efficacy signal.

### Secondary outcomes

#### Depth of haematologic response

Rates of other IMWG response categories at T_1_, including very good partial response (VGPR), partial response (PR), and stable disease (SD), in each arm. Stringent CR (sCR) will also be noted.

#### Survival outcomes

We will record PFS and OS during and after the trial. PFS is defined from randomisation to disease progression, relapse, or death, and OS from randomisation to death from any cause. Given the study’s design, these data will be descriptive.

#### Patient nutritional status and dietary adequacy

Changes in body weight, BMI, waist circumference, NRS-2002 score, and body composition (phase angle via bioelectrical impedance analysis) will be tracked from baseline through treatment and follow-up. Laboratory markers of nutritional status (albumin) and metabolic biomarkers (glucose, ketones, insulin, and IGF-1) will be measured at baseline and T_1_.

#### Safety and tolerability

The incidence of AEs and serious adverse events (SAEs) will be compared between arms, with attention to ≥ grade 3 according to the NCIC Common Terminology Criteria for Adverse Events (CTCAE) AEs. We will examine if the TRE arm has any increase in events like dehydration, electrolyte imbalances, infections, or other complications.

#### Quality of life (QoL)

Patient-reported outcomes will be measured using the EORTC QLQ-C30 questionnaire at baseline, end of treatment, and end of follow-up.

#### Fatigue

Fatigue will be assessed by the FACIT-fatigue scale (Functional Assessment of Chronic Illness Therapy-Fatigue).

#### Exploratory biological endpoints

The trial will collect biological samples to investigate mechanistic effects of TRE. BM and blood specimens at baseline and end of treatment will be analysed for changes in MM cell biology and metabolism. These include examining clonal evolution and gene expression in MM cells, shifts in immune cell profile in marrow, and levels of metabolic hormones. Serum and plasma will be used for exploratory metabolomic and proteomic analyses to generate hypotheses on the biological impact of TRE. The microbiome has been linked to cancer pathologies and treatment [22]. It largely contributes to immune system maturation [31] and diet is a main driver of both microbiome composition and function [29]. Thus, TRE-induced microbiome changes could contribute to treatment outcome. Microbiome composition will be monitored using metagenomic sequencing.

## Sample size

The sample size for this trial was based on precision for the adherence endpoint, not statistical power for efficacy. We set a target that ≥ 80% of patients in the TRE arm must achieve adequate adherence for the intervention to be deemed feasible. Using a one-sample proportion calculation, enrolling 27 patients in the experimental arm allows the adherence rate to be estimated with a margin of error of approximately ± 15% at 95% CI. Therefore, 54 patients were chosen to assess feasibility with adequate precision. This sample also matches typical pilot trial sizes in oncology and is sufficient to identify major safety or compliance issues.

## Randomisation and blinding

Eligible patients are randomised immediately after screening using permuted blocks to ensure unpredictability. Allocation concealment is maintained via a secure web-based randomisation module in the electronic data capture system.

The trial is not blinded. Participants know their group assignment as they must actively follow or not follow a fasting schedule, and study clinicians also know which arm a patient is in. Treating physicians are instructed to manage patients uniformly according to clinical guidelines and not alter therapy based on arm assignment. Outcome assessors for survival and disease progression are inherently unblinded since they are involved in patient care, but progression is determined by IMWG criteria to maintain consistency.

An independent DMC is instituted to provide oversight of safety and trial conduct. The DMC is unblinded and will review interim safety data. They will monitor AE rates in both arms, focusing on harm signals in the TRE group. The DMC is empowered to recommend protocol modifications or early trial termination if unacceptable risks or futility are observed.

## Statistical methods

All analyses will follow the ITT principle. A per-protocol (PP) analysis will be performed as a sensitivity analysis. Safety analyses will include all patients who started the intervention or control and may be stratified by actual fasting behaviour if crossovers occur.

For the primary endpoint feasibility, the adherence rate in arm A will be calculated as patients with ≥ 80% fasting-day compliance divided by total patients in arm A, with a 95% CI (Clopper-Pearson). No hypothesis test is needed for this single-arm feasibility measure, and we will compare the observed adherence to our target threshold.

For the preliminary efficacy endpoint of CR with MRD negativity at T_1_, proportions between both arms will be compared using a chi-square or Fisher’s exact test to explore differences. We will estimate the absolute difference in CR/MRD rates between arm A and B with CI. To adjust for potential imbalances and our stratification factors, we will perform logistic regression: The dependent variable will be CR/MRD-negative status, and independent variables will include treatment arm, line of therapy, baseline BMI category, and gender. The logistic regression will provide an adjusted OR with a 95% CI for achieving CR/MRD negativity, yielding a hypothesis-generating effect size. We will examine MRD negativity alone and CR (regardless of MRD) separately as post hoc analyses.

Secondary endpoint analysis will be descriptive and exploratory: Response categories (VGPR, PR, SD) will be tabulated by arm. Survival endpoints (PFS, OS) will be plotted using Kaplan–Meier curves. Continuous measures like weight, BMI, and lab values will be analysed using mixed-effects models or paired t-tests within arms and compared between arms by analysis of covariance (ANCOVA), adjusting for baseline. AEs will be summarised by category and grade. QoL and fatigue scores will be analysed by calculating changes from baseline to T_1_. Mean changes will be compared between arms using t-tests or non-parametric tests if data are not normally distributed. Exploratory biological endpoints will be analysed for hypothesis-generation.

We anticipate minimal missing data for primary endpoints since adherence can be assessed if diary data are > 80% complete. Participants who discontinue TRE or withdraw before adherence can be evaluated will be counted as non-adherent for the feasibility endpoint, and intervention pauses and reasons will be summarised descriptively. If a patient lacks MRD status at T_1_, they will be considered not achieving CR/MRD. Missing data in secondary patient-reported outcomes will be handled by analysing available cases or using imputation methods. For survival, standard censoring at last follow-up is done for those without events. A sensitivity analysis using multiple imputation for missing outcome data will be explored to ensure robustness.

All statistical tests will be two-sided, with *p*-value < 0.05 considered significant for exploratory, hypothesis-generating purposes. No adjustments for multiple comparisons are planned, given the exploratory nature.

## Ethics and dissemination

The protocol was approved by the Ethics Committee of the University of Lübeck (2024-182_1) and will be conducted in line with Declaration of Helsinki and ICH-GCP. The study is registered in the German Clinical Trials Register (DRKS00034370). All participants will provide written informed consent as well as broad consent for molecular analyses and may withdraw at any time. Participant data will be handled under EU General Data Protection Regulation and national law. Safety will be overseen by an independent DMC, which will review interim data and may recommend modifications or early termination.

Results will be submitted for publication irrespective of outcome, following CONSORT guidance for randomised trials. Participants will receive a lay summary and may request their individual outcomes. If TRE proves safe, this protocol will inform a larger phase II trial.

## Discussion

FASTINA is the first randomised feasibility study examining 16:8 TRE in MM patients during active therapy. The central question is whether selected MM patients – despite treatment toxicities, steroid-associated metabolic changes, and nutritional challenges – can maintain a daily eating-window intervention without compromising caloric and protein intake. Prior short-term fasting studies in solid malignancies suggest feasibility in some settings [3, 8, 10], but evidence is heterogeneous. In the DIRECT trial, adherence declined over repeated chemotherapy cycles and severe toxicity, and pathological complete response were not significantly improved in the ITT population [11]. Therefore, FASTINA will monitor adherence, dropout, screen-failure reasons, and non-adherence reasons as central feasibility outcomes rather than assuming broad applicability.

Patient safety is paramount and ensured by multiple safeguards, including stringent exclusion criteria, nutritional counselling, DMC oversight, and predefined discontinuation thresholds. We anticipate that TRE can be feasible in well-nourished MM patients, but potential risks include weight loss, inadequate protein intake, sarcopenia, dehydration, electrolyte disturbances, infection-related anorexia, and treatment-related gastrointestinal toxicity. Any signal for toxicity reduction will be interpreted cautiously given the pilot design. A unique consideration in MM is the use of high-dose dexamethasone. Dexamethasone may induce hyperphagia, hyperglycaemia, and circadian metabolic disruption, potentially masking or counteracting fasting-associated metabolic changes [14]. Steroid exposure and biomarker timing will therefore be documented and explored rather than interpreted as pure TRE effects.

While not powered for definitive efficacy, FASTINA’s exploratory outcomes could be hypothesis-generating. Preclinical evidence suggests that tumour cells exposed to nutrient deprivation may demonstrate increased apoptosis when treated with cytotoxic drugs [6, 21]. However, clinical data do not yet support a definitive claim that TRE deepens response or converts MRD status in MM. CR/MRD endpoints are included to estimate effect sizes and variability for a future trial. Our exploratory biomarker analyses – evaluating clonal evolution and metabolomic profiles – may offer mechanistic insights but will require validation.

This study has limitations common to pilot trials [32]. The single-centre design, anticipated slow recruitment, small sample size, and treatment heterogeneity could dilute observable effects and will preclude definitive efficacy conclusions. We enrol both newly diagnosed and relapsed patients, which introduces variability in prognoses and treatments. Additionally, the open-label design could introduce bias from placebo effects or behavioural differences in self-reported outcomes. We equalised contact by providing counselling to both arms, but some bias is inevitably possible. Food diaries are vulnerable to reporting bias, therefore, diary data will be interpreted together with body composition and metabolic biomarkers, while acknowledging that these objective measures are not specific for TRE adherence. Another limitation is the short intervention duration of 3 months – chosen to coincide with typical induction therapy length and to mitigate dropout – which may not be sufficient to influence long-term outcomes like PFS [32]. It will, however, capture initial response. We also acknowledge that our patient population is selected by design – excluding those with frailty, significant weight loss, cachexia or malnutrition – so results may not generalise to all MM patients, especially those who are older or have poor nutritional status [4].

If FASTINA demonstrates feasibility, acceptable safety, and a preliminary response signal, a larger, multicentre phase II trial focusing on efficacy with response rate or PFS as primary endpoint should follow. It could incorporate refinements from FASTINA, such as optimal fasting timing, objective adherence tools, patient education strategies to improve adherence, and robust patient-reported outcome measures. In parallel, the biological samples from FASTINA may yield insights that open new research questions – for instance, if fasting is found to substantially reduce IGF-1 during therapy, one could investigate combining TRE with drugs targeting the IGF-1 pathway [27]. Or if TRE appears to improve T-cell counts or function, it might synergise with immunotherapies like checkpoint inhibitors or CAR-T cells – a hypothesis for future trials [16, 17, 24, 34, 35].

In conclusion, FASTINA represents an initial, safety-focused step in translating metabolic therapy modulation to haematological cancers. A successful outcome – high adherence, preserved nutritional status, no excess toxicity, and a hypothesis-generating efficacy signal – would justify definitive testing. Only larger adequately powered studies can determine whether TRE should become part of supportive MM care. The knowledge gained will contribute to onco-metabolism and may inform future MM management.

## Supplementary Information

Supplementary Material 1: Supplementary Fig. 1. SPIRIT figure. Figure showing timepoints of enrolment, intervention and assessments.

## Data Availability

Data are available upon request.

## Abbreviations

AE: Adverse event
AKT: Protein kinase B
ANCOVA: Analysis of covariance
ASCT: Autologous stem cell transplantation
BIA: Bioelectrical impedance analysis
BM: Bone marrow
BMI: Body mass index
CAR T: Chimeric antigen receptor T cell
CI: Confidence interval
CONSORT: Consolidated Standards of Reporting Trials
CR: Complete response
CTCAE: Common Terminology Criteria for Adverse Events
DMC: Data Monitoring Committee
DRKS: German Clinical Trials Register
EORTC QLQ-C30: European Organisation for Research and Treatment of Cancer Quality of Life Questionnaire – Core 30
EU: European Union
FACS: Fluorescence-activated cell sorting
FACIT: Functional Assessment of Chronic Illness Therapy
FACIT-fatigue: Functional Assessment of Chronic Illness Therapy – Fatigue
FACT-G: Functional Assessment of Cancer Therapy – General
GCP: Good Clinical Practice
GDPR: General Data Protection Regulation
HIV: Human immunodeficiency virus
ICH-GCP: International Council for Harmonisation – Good Clinical Practice
IGF-1: Insulin-like growth factor 1
IMiDs: Immunomodulatory drugs
IMWG: International Myeloma Working Group
mABs: Monoclonal antibodies
MRD: Minimal residual disease
mTOR: Mechanistic target of rapamycin
NGS: Next-generation sequencing
NRS-2002: Nutritional Risk Screening 2002
OR: Odds ratio
OS: Overall survival
PFS: Progression-free survival
PI3K: Phosphoinositide 3-kinase
PIs: Proteasome inhibitors
PR: Partial response
QoL: Quality of life
ROS: Reactive oxygen species
SAE: Serious adverse event
sCR: Stringent complete response
SD: Stable disease
SoC: Standard of care
T_0_: Screening
T_1_: End of treatment
T_2_: End of follow-up
TCA: Tricarboxylic acid cycle
TRE: Time-restricted eating
VGPR: Very good partial response
