# Supplementary material for: FASTINA – protocol for a randomised feasibility trial of time-restricted eating to improve response to multiple myeloma therapy
Source: Pilot Feasibility Stud. 2026 Sep 3;12:130. doi: 10.1186/s40814-026-01914-7 (PMC13540832; doi:10.1186/s40814-026-01914-7)
Supplement: Supplementary file 1 [file 40814_2026_1914_MOESM1_ESM.docx]

**9.3	Supplementary information**

|  | **TRIAL PERIOD** |  |  |  |
| --- | --- | --- | --- | --- |
|  | **Enrollment** | **Post-randomization** | | **Close-out** |
| **TIMEPOINT** | ***-3 – 0 m*** | **0 m** | ***3 m*** | ***6 m*** |
| **ENROLLMENT:** |  |  |  |  |
| **Eligibility screen** | X |  |  |  |
| **Informed consent** | X |  |  |  |
| **Randomization** |  | X |  |  |
| **INTERVENTION/ COMPARATOR:** |  |  |  |  |
| ***TRE*** |  | X |  | X |
| **ASSESSMENTS:** |  |  |  |  |
| ***Nutritional status, BIA, BM biopsy etc. (see Fig. 2)*** | X | X |  |  |
| ***Adherence, therapy response (see 3.1)*** |  |  | X | X |
| ***Metabolic biomarker measurement*** |  | X | X |  |
| ***OS, PFS, safety, CTCAE, QoL etc. (see 3.2)*** |  |  | X | X |

***Supplementary Figure 1. SPIRIT figure.*** *Figure showing timepoints of enrolment, intervention and assessments.*
